# Voxel-Based Analysis of [18F]-FDG Brain PET in Rats Using Data-Driven Normalization

**DOI:** 10.3389/fmed.2021.744157

**Published:** 2021-10-20

**Authors:** Silke Proesmans, Robrecht Raedt, Charlotte Germonpré, Emma Christiaen, Benedicte Descamps, Paul Boon, Veerle De Herdt, Christian Vanhove

**Affiliations:** ^1^4Brain Lab, Department of Head and Skin, Ghent University, Ghent, Belgium; ^2^IbiTech-MEDISIP-Infinity Lab, Department of Electronics and Information Systems, Ghent University, Ghent, Belgium

**Keywords:** [18F]-FDG, PET, intensity normalization, voxel-based analysis, rat brain

## Abstract

**Introduction:** [18F]-FDG PET is a widely used imaging modality that visualizes cellular glucose uptake and provides functional information on the metabolic state of different tissues *in vivo*. Various quantification methods can be used to evaluate glucose metabolism in the brain, including the cerebral metabolic rate of glucose (CMR_glc_) and standard uptake values (SUVs). Especially in the brain, these (semi-)quantitative measures can be affected by several physiological factors, such as blood glucose level, age, gender, and stress. Next to this inter- and intra-subject variability, the use of different PET acquisition protocols across studies has created a need for the standardization and harmonization of brain PET evaluation. In this study we present a framework for statistical voxel-based analysis of glucose uptake in the rat brain using histogram-based intensity normalization.

**Methods:** [18F]-FDG PET images of 28 normal rat brains were coregistered and voxel-wisely averaged. Ratio images were generated by voxel-wisely dividing each of these images with the group average. The most prevalent value in the ratio image was used as normalization factor. The normalized PET images were voxel-wisely averaged to generate a normal rat brain atlas. The variability of voxel intensities across the normalized PET images was compared to images that were either normalized by whole brain normalization, or not normalized.

To illustrate the added value of this normal rat brain atlas, 9 animals with a striatal hemorrhagic lesion and 9 control animals were intravenously injected with [18F]-FDG and the PET images of these animals were voxel-wisely compared to the normal atlas by group- and individual analyses.

**Results:** The average coefficient of variation of the voxel intensities in the brain across normal [18F]-FDG PET images was 6.7% for the histogram-based normalized images, 11.6% for whole brain normalized images, and 31.2% when no normalization was applied. Statistical voxel-based analysis, using the normal template, indicated regions of significantly decreased glucose uptake at the site of the ICH lesion in the ICH animals, but not in control animals.

**Conclusion:** In summary, histogram-based intensity normalization of [18F]-FDG uptake in the brain is a suitable data-driven approach for standardized voxel-based comparison of brain PET images.

## Introduction

[18F]-fluorodeoxyglucose positron emission tomography ([18F]-FDG PET) is increasingly used for the diagnosis, staging, prognosis, and response evaluation of a variety of diseases (1, 2). Especially in the brain, evaluation of the global and regional cerebral glucose consumption provides essential information on brain function and metabolism in neurological disorders. PET image interpretation in clinical practice is mainly performed by visual inspection, but an increasingly important role has been established for (semi-)quantitative image analysis, for example by standard uptake values (SUVs) or the cerebral metabolic rate of glucose (CMR_glc_) (3).

In general, PET (semi-)quantification methods commonly rely on region of interest (ROI)-based or voxel-wise statistical comparison between PET scans of patients and healthy subjects (4). Both methods have their limitations and difficulties. ROI-based analysis can be biased by the choice of the ROI, while voxel-based analysis is less biased but requires a large pool of healthy subjects. Another challenging issue related to (semi-)quantitative brain PET analysis is that uptake of [18F]-FDG in the brain poses a large degree of variability due to physical or biological factors, and inconsistent image acquisition, processing and analysis (5). Especially in the brain, [18F]-FDG uptake, and thus measures such as SUVs, are affected by several physiological factors, including blood glucose level, age, gender, circadian rhythm, and stress (6, 7). Therefore, intensity normalization of the PET signal is recommended for comparing [18F]-FDG uptake in ROIs or voxel values in the brain (8).

Intensity normalization is usually performed by scaling voxel intensities based on the average uptake in a previously defined reference region where tracer uptake is not affected by the studied disease, or based on a data-driven approach where the average uptake in the whole brain is the simplest and most used method. However, these methods might introduce bias depending on the choice of reference region or the presence of regional uptake changes, respectively (8, 9). The lack of standardization of intensity normalization procedures has been recognized as a major weakness for the harmonization of (semi-)quantitative [18F]-FDG PET analysis (10).

The effect of different normalization procedures on lesion visualization was investigated in a recent paper by López-González et al. (8) using simulated data. Data-driven methods, and the histogram-based intensity normalization method in particular, seemed to introduce the least bias when performing voxel-wise statistical analysis on PET data. In this paper, a workflow will be presented to create a normal [18F]-FDG rat brain atlas using histogram-based intensity normalization.

Additionally, the same intensity normalization approach will be used to detect changes in glucose metabolism in a rat model for intracerebral hemorrhage (ICH). The nature and mechanisms of glucose uptake changes post-ICH are outside the scope of this article. However, the use of a normal brain PET atlas has been proven useful for the analysis of extensive as well as subtle metabolic changes in the brain in pathological circumstances (11–13). Therefore, the feasibility of using the histogram-based intensity normalization procedure in subsequent voxel-wise statistical comparison of post-ICH [18F]-FDG PET images with the normal rat brain atlas will be demonstrated in this paper.

## Methods

### Animals

Twenty-eight healthy adult male Sprague-Dawley rats (Envigo, The Netherlands) of 11 weeks old and with an average weight of 317.4 ± 25.7 g were included in this study for the generation of the normal rat brain atlas. Later, 18 of these 28 animals were used in further experimental procedures. In 9 of these animals an ICH was induced, and the 9 other animals were used as a saline control for subsequent voxel-wise comparison of ICH or control PET scans with the normal brain atlas. Animals were individually housed under controlled conditions (12/12 h light/dark cycle, temperature 20–24°C and relative humidity 40–60%) and handled over a period of 6 days for 10 min per day to reduce stress during the experimental procedures. Animals received food and water *ad libitum* and were fasted during the nights before [18F]-FDG PET scanning. The animals were treated according to the European guidelines (directive 2010/63/EU) and the protocol was approved by the local Ethical Committee on Animal Experiments of Ghent University (ECD 19/80).

### Scanning Procedures

Animals were food deprived for at least 12 h before tracer injection to lower their blood glucose level (6), so that [18F]-FDG would not compete with endogenous glucose uptake via GLUT-transporters in the brain. 32.5 ± 7.8 MBq [18F]-FDG was administered intravenously into one of the lateral saphenous tail veins under short anesthesia (2% isoflurane and oxygen mixture). Immediately afterwards, animals were awakened and placed in a heated cage, to reduce tracer uptake in brown fat. The cage was placed inside a dark room, to minimize uptake into the Harderian glands, reducing spill-over effects from these glands to the brain.

After 60 min of tracer uptake, a 15 min static PET scan was acquired (β-cube, Molecubes NV, Ghent, Belgium) under general anesthesia (2% isoflurane and oxygen mixture) and using a heated animal bed. PET data were iteratively reconstructed with Molecubes β-Cube software (Version 1.5.7) by an Ordered Subset Expectation Maximization (OSEM) algorithm using 30 iterations and 1 subset into a 196 × 196 × 384 matrix with a voxel size of 400 μm. An energy window of 30% centered on the 511 keV photopeak was used. The voxel values in these reconstructed images were expressed as kBq/cc and were converted to SUVs. SUVs were voxel-wise calculated as follows: $\frac{C_{i}}{D/W}$, where *C* represents the radioactivity concentration (kBq/cc) measured by the PET scanner in voxel *i*, *D* is the decay-corrected injected [18F]-FDG activity (kBq) and *W* is the weight of the animal (*g*).

Afterwards, TurboRARE T2-weighted magnetic resonance imaging (MRI) was performed on a 7T system (PharmaScan 70/16, Bruker, Germany) under general anesthesia using a transmit/receive volume coil with 40 mm inner diameter (Bruker, Germany). A circulating-water heating pad and a pressure sensor were used to maintain the animals' body temperature and monitor their respiratory rhythm, respectively. After optimizing the magnetic field homogeneity the following acquisition parameters were used: repetition time 3,700 ms, echo time 37 ms, in-plane slice resolution 109 × 109 μm^2^, 30 contiguous slices of 600 μm thickness, matrix size 320 × 320, 4 averages, resulting in a total acquisition time of 9 min.

### Normal Rat Brain Atlas

#### Pre-processing

The reconstructed PET images were preprocessed using a four-step procedure: converting, cropping, filtering and coregistration. First, the reconstructed PET DICOM images were converted to NIfTI format using the MRtrix3 (14) command *mrconvert*. In the second preprocessing step, images were cropped into a 80 × 80 × 80 matrix (32 × 32 × 32 mm field-of-view) to only visualize the brain using the MRtrix3 command *mrcrop*. The cropped images were then smoothed by a Gaussian filter with a full width at half maximum (FWHM) of 1 mm using the MRtrix3 command *mrfilter*. Finally, all converted, cropped and filtered PET images were geometrically aligned by non-linear coregistration using the *population_template* command in MRtrix3, which generated an initial brain atlas template by voxel-wisely averaging all coregistered images (*n* = 28).

#### Normalization Procedures

The acquired, reconstructed, converted, cropped, filtered, and coregistered images were normalized via different procedures in order to compare the variability of voxel intensities in the brain across the normalized PET images. After each normalization method, voxel-wise calculations of the mean and standard deviation across the images were performed by *mrmath* in MRTrix3. Next, coefficient-of-variation (CoV) maps were generated by voxel-wise calculation of: $\sigma_{i}/\mu^{*}100\%$, with μ_μ_ and σ_σ_ being the mean and standard deviation across all normalized PET images in voxel *i*.

##### Data-Driven Histogram-Based Normalization

Each individually converted, cropped, filtered, and coregistered PET image was voxel-wisely divided by the initial brain atlas template using the MRtrix3 command *mrcalc*. The resulting ratio image was used to generate a histogram of the voxel-wisely calculated ratios, only including ratios within the normal brain region. The normal brain region was defined as all voxels with voxel intensities larger than 50% of the maximum [18F]-FDG brain uptake on the initial brain template.

The maximum of the histogram, which represents the most prevalent ratio in the brain, was used as normalization factor. Each individually converted, cropped, filtered, and coregistered PET image was divided by this normalization factor using *mrcalc*. Subsequently, the final normal [18F]-FDG rat brain atlas was generated by voxel-wisely calculating the average and standard deviation of all histogram-based normalized PET images using *mrmath*.

##### Global Mean Scaling

Global mean scaling, or whole-brain normalization, was performed by scaling the images to the average [18F]-FDG uptake value in the whole brain. The whole brain region was defined as all voxels with voxel intensities larger than 50% of the maximum [18F]-FDG brain uptake on the initial brain template. The mean SUV value inside this brain region was calculated for each PET image individually and used as normalization factor.

##### No Normalization

Lastly, the converted, cropped, filtered, and coregistered images were further analyzed without performing any normalization and with voxel values expressed as SUVs.

### ICH Animal Model

#### ICH Induction

In nine animals, an ICH was induced by striatal injection of 0.6U collagenase (type VII-S, C2399, Sigma-Aldrich) dissolved in 0.7 μl saline (AP 0.5, ML 3.5, DV 6.0 mm relative to Bregma). Nine other animals were injected with 0.7 μl saline as a control. The injections were performed under general anesthesia (2% isoflurane and oxygen mixture) using a Neuros-Syringe (model 7001, point style 4, Hamilton) and a Quintessential Stereotaxic Injection system (Stoelting, IL, USA), at a flowrate of 0.14 μl/min. After injection, the syringe was left in place for an additional 5 min to prevent backflow.

#### Preprocessing and Histogram-Based Normalization

One day after inducing ICH, [18F]-FDG PET scans were acquired using the same imaging procedure as described above. The acquired PET scans were also preprocessed as described above. After reconstruction, conversion to NIfTI format, cropping, 1 mm Gaussian filtering and coregistration on the generated normal rat brain atlas, ratio images were calculated by voxel-wisely dividing the individual post-ICH or post-saline [18F]-FDG PET images by the normal rat brain atlas template. The post-ICH or post-saline images were then normalized using the most prevalent ratio within the normal brain region obtained from the normal rat brain atlas.

### Voxel-Wise Analysis

#### Group Analysis

After preprocessing and normalization, post-ICH (*n* = 9) or post-saline injection (*n* = 9) images were voxel-wisely compared to the normal [18F]-FDG rat brain images (*n* = 28) using group-level analysis. Voxel-wise *t*-test statistics with False Discovery Rate (FDR) based correction for multiple comparisons (*p* < 0.05) were calculated in the Statistical Parametric Mapping software tool (SPM12 version 7771). The results of this group analysis were overlayed on T2-weighted average templates of the ICH or control animals, which were generated by the *popultion_template* command in MRtrix3. Overlays were made with PMOD software (PMOD Technologies version 3.405, Zürich, Switzerland).

#### Individual Analysis

Individual histogram-based normalized images of ICH and control animals were transformed into *Z*-score maps by voxel-wise calculation of *Z*-score statistics: $z\;={\frac{x_{i}-\mu_{i}}{\sigma }}_{i}$, where *u* and σ represent the mean and standard deviation of voxel *i* in the normal database and *x* is the voxel value of voxel *i* in the coregistered and normalized PET scan of an ICH or control animal. A 95% confidence interval was calculated for all *Z*-scores in the brain and *Z*-score maps were subsequently thresholded to only show *Z*-scores outside of this confidence interval, representing the 2.5% lowest and 2.5% highest values in the images. The final thresholded *Z*-score images were overlayed on T2-weighted average templates in PMOD.

## Results

### Histogram-Based Normalization

**Figure 1:**
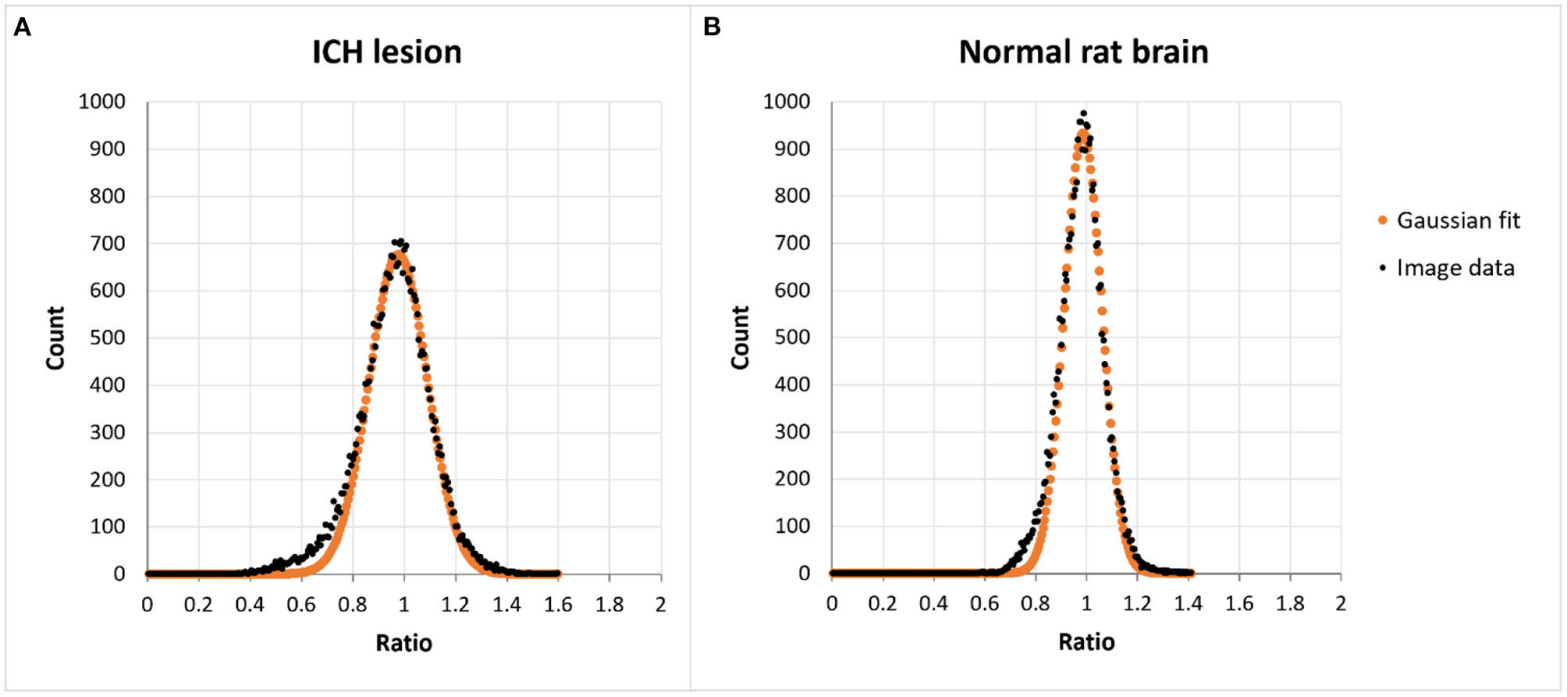
Histograms of the voxel values present in ratio-images obtained by voxel-wise dividing a PET image with the normal rat brain atlas template obtained from **(A)** an animal with an ICH lesion and **(B)** a normal [18F]-FDG rat brain scan. In animals with a lesion there is a higher prevalence of lower ratios without affecting the peak position.

As described above, histograms of ratios, obtained by voxel-wisely dividing [18F]-FDG PET images by the normal rat brain atlas template, were generated for the calculation of a normalization factor. Figure 1 shows the histograms obtained from a post-ICH (A) and of a normal (B) [18F]-FDG PET image. Black dots represent the prevalence of a particular ratio, whereas the orange dots represent a Gaussian curve that fits through these histogram data.

The image of the ICH animal shows somewhat higher prevalence of lower ratios (0.4–0.8) and lower counts around the maximum of the histogram as compared to the normal image. The peak of the histogram, representing the most prevalent ratio, is centered around the value of one in both images.

### [18F]-FDG Normal Rat Brain Atlas

**Figure 2:**
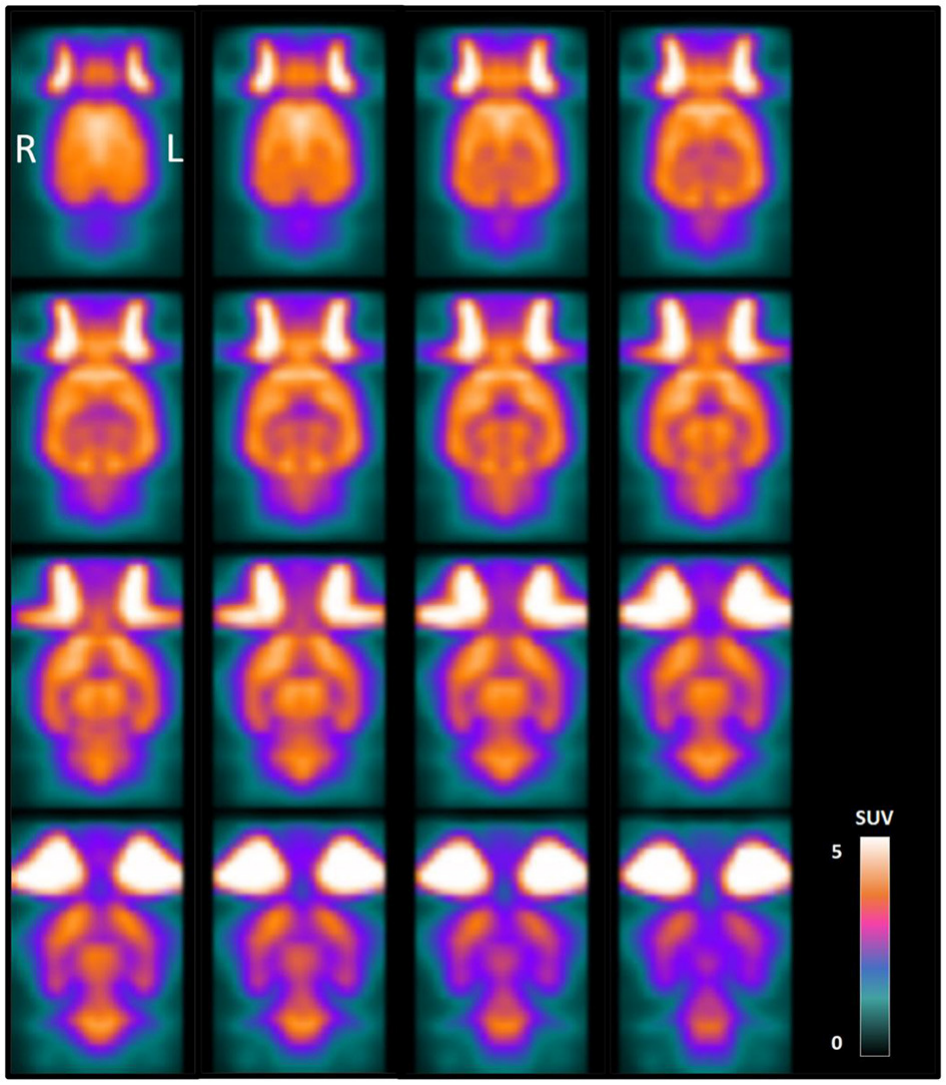
Normal rat brain PET atlas obtained by averaging the reconstructed [18F]-FDG PET images of 28 healthy adult male Sprague-Dawley rats after cropping, Gaussian filtering, coregistration and histogram-based intensity normalization. Coronal slices are shown from dorsal (top left) to ventral (bottom right) and voxel values are presented in SUVs.

**Figure 3:**
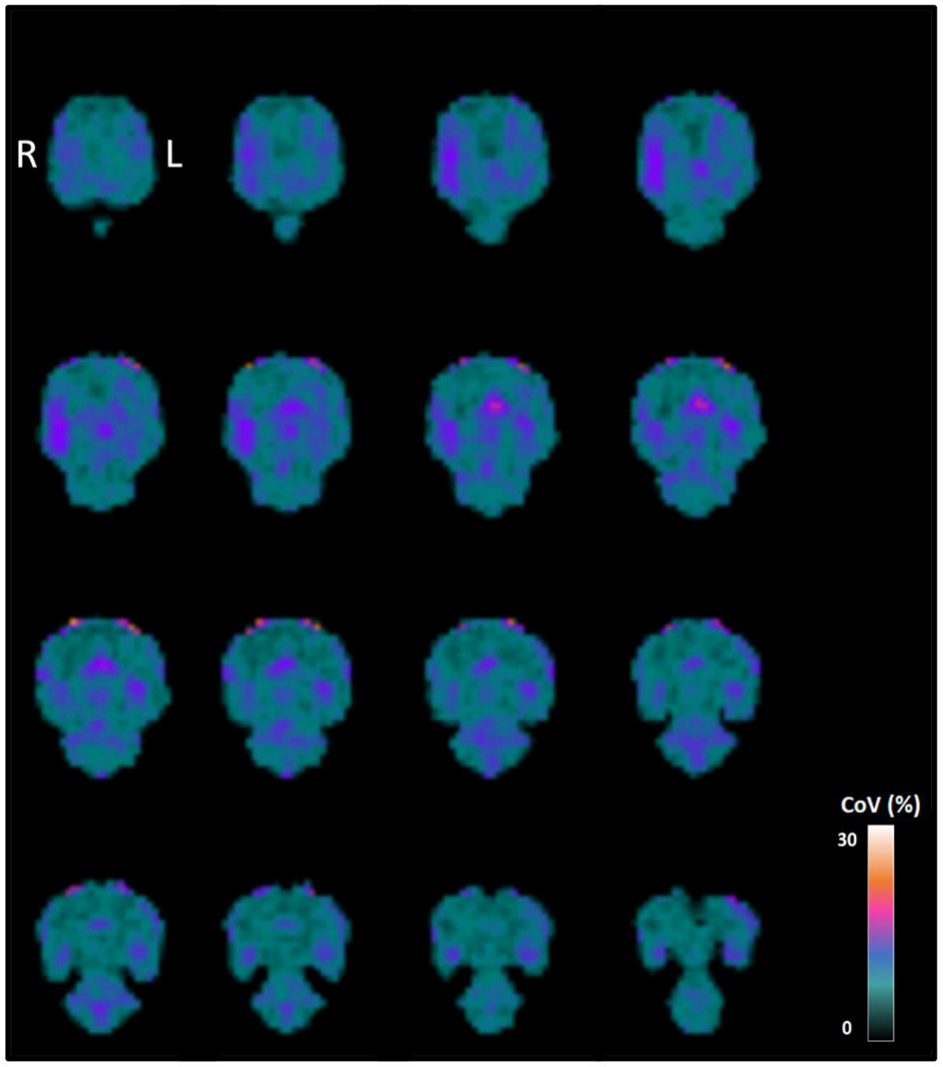
Between-subject coefficient of variation obtained by voxel-wise calculating the ratio of the standard deviation to the mean of the [18F]-FDG images from the 28 healthy adult male Sprague-Dawley rats used to generate the normal brain template. Coronal slices are shown from dorsal (top left) to ventral (bottom right).

The generation of a histogram-based normalized normal brain atlas was feasible and straightforward using MRTrix3 software. Figure 2 shows the normal rat brain atlas after histogram-based intensity normalization. Voxel-wise coefficients of variation in this atlas, representing the relative variability between voxel values across all images in the normal rat brain atlas, are shown in Figure 3.

As shown in Table 1, the mean CoV in the brain after histogram-based normalization was 6.71 ± 2.05%, which is 78% lower than when the images were not normalized. In the latter case, when non-normalized SUV images were used to generate a normal rat brain atlas, the mean CoV in the brain was 31.17 ± 1.54%. The mean CoV after histogram-based normalization was 42% lower compared to the whole brain normalization method (mean CoV: 11.61 ± 1.41%).

**Table 1:** Mean CoV values in the brain for the different PET templates.

**Normalization method**	**CoV (%)**
Histogram-based	6.71 ± 2.05
Global mean scaling	11.61 ± 1.41
No normalization	31.17 ± 1.54

### Voxel-Wise Statistical Analysis

**Figure 4:**
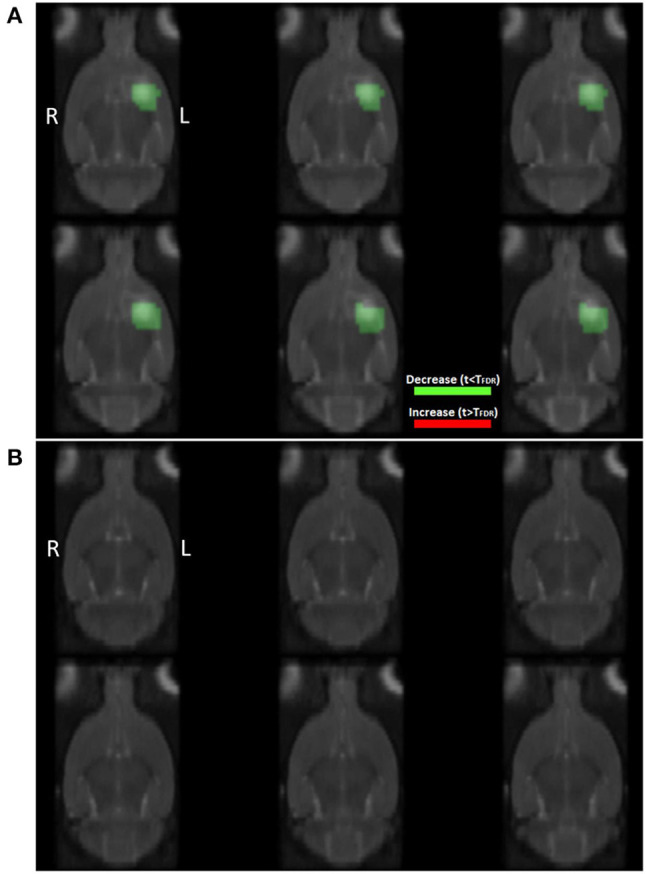
Coronal slices showing the results of statistical analysis on the group level using *t*-test statistics with correction for multiple comparisons when comparing **(A)** [18F]-FDG PET images of ICH animals (*n* = 9), and **(B)** [18F]-FDG PET images of control animals (*n* = 9), to the normal [18F]-FDG PET rat brain atlas (*n* = 28). T_FDR_: threshold calculated in SPM using False Discovery Rate based correction for multiple comparisons (*p* < 0.05). Red: significantly increased glucose uptake (not visible in this image), green: significantly decreased glucose uptake. Colored regions are overlayed on the interpolated T2 templates of ICH and control animals at day 1 post-injection, respectively.

The PET images of nine ICH animals and nine control animals were preprocessed and normalized in the same way as the images of the normal brain atlas. Statistical voxel-wise comparisons of [18F]-FDG images post-ICH with the normal rat brain atlas were performed on a group level to visualize regions of significantly increased or decreased glucose uptake after correction for multiple comparisons. Figure 4 shows the resulting images of this group analysis after histogram-based normalization. The analysis clearly shows a region of significantly decreased glucose uptake at the site of the ICH lesion (Figure 4A). No significant regions were observed when comparing [18F]-FDG images of control animals to the normal rat brain atlas (Figure 4B).

**Figure 5:**
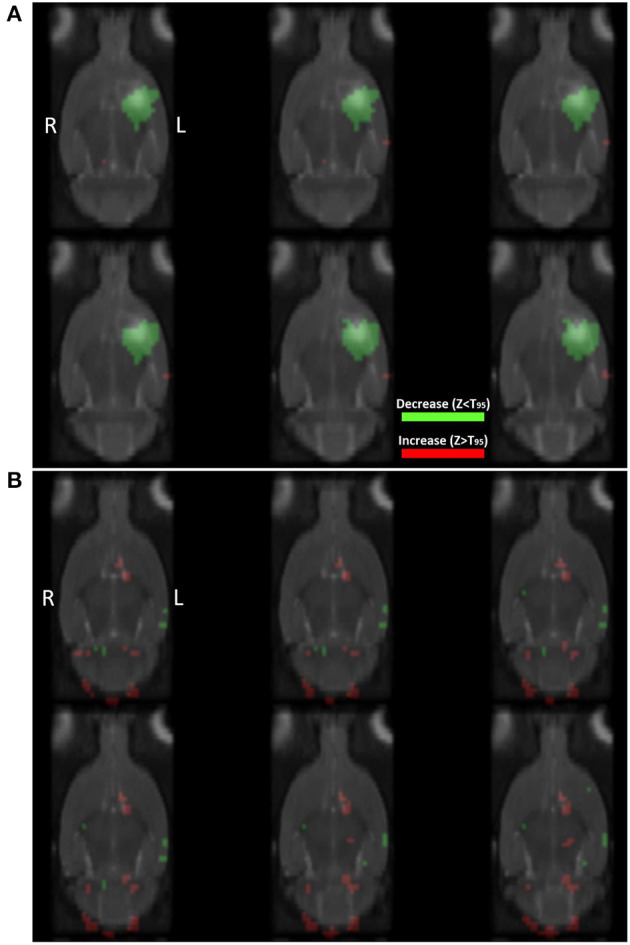
Coronal slices showing the results of statistical analysis on the individual level by voxel-wise calculation of z-scores using **(A)** an ICH animal and **(B)** a control animal. T_95_: threshold based on the 95% confidence interval of *Z*-scores inside the brain. Red: significantly increased glucose uptake, green: significantly decreased glucose uptake compared to baseline. Colored regions are overlayed on the interpolated T2 templates of ICH and control animals at day 1 post-injection, respectively.

The generation of *Z*-score maps and subsequent thresholding by a 95% confidence interval, resulted in the visualization of regions with significantly increased or decreased glucose metabolism compared to the normal rat brain template on an individual level. Figure 5 shows the thresholded *Z*-map of an ICH animal (A) and of a control animal (B) overlayed on the corresponding T2 templates. In the ICH animal, a clear region of decreased glucose uptake is visible at the site of the lesion. In the control animal, no such regions of significantly increased or decreased glucose uptake are visible. Instead, the voxels representing the lowest 2.5% and highest 2.5% of voxel values in the brain, are sparsely distributed across the brain, as would be expected.

## Discussion

The goals of this study were 2-fold: (1) to present a workflow for the creation of a normal [18F]-FDG PET rat brain atlas based on a data-driven histogram-based approach for intensity normalization, and (2) to use these data-driven normalized data in a voxel-wise statistical analysis to detect significant changes in glucose uptake in a rat model for ICH when compared to the normal rat brain template. As our data shows, histogram-based intensity normalization seems to be an accurate approach to detect changes in glucose metabolism in the rat brain using a normal database.

Recent ground truth-based research by Lopéz-González et al. (8) investigated the effect of different PET normalization methods on the detection of simulated hypometabolic patterns. As the authors of this study have shown, under- or overestimation of the normalization factor can lead to significant differences in PET interpretation, demonstrating the importance of accurate and appropriate use of intensity normalization methods. Indeed, normalization is essential for accurate PET analysis, which was clearly demonstrated by the high CoV (31.17 ± 1.54%) in our analysis when no normalization was performed on the imaging data.

The high CoV across non-normalized SUV images might lie in the fact that the SUV measure is highly influenced by inter- and intra-variable factors. SUVs are calculated as the measured radioactivity concentration in a voxel multiplied with the subject's weight and divided by the decay-corrected injected tracer dose (5). However, [18F]-FDG uptake in the brain is affected by many other factors, besides the injected dose and subject's weight. For example, biological factors such as blood glucose level, gender and age, and methodological issues such as scanner calibration and tracer spill or backflow while injecting, may severely influence SUV-based quantification (15–17). Several SUV correction methods have been used to decrease inter- and intra-animal variability, such as correction for pre- or post-scan blood glucose level or serum corticosterone concentration (6). Still, inter-animal CoV varies between 9.57 and 69.11% depending on the SUV correction factor that is used (6), again indicating the influence of different PET normalization or quantification methods on result interpretation.

Common intensity normalization methods include the use of a reference region or data-driven approaches. Regions such as the cerebellum, pons, white matter and primary sensorimotor cortex have been used as reference region in different types of diseases (9, 18, 19). However, normalization based on a reference could be biased by the choice of the reference area, which should not be affected by the disease that is studied (8, 9). Thus, the size and location of a reference region should be very carefully chosen depending on the disease state. One of the most widely employed methods of data-driven normalization is global mean scaling, where the average brain uptake value is used as reference. Depending on the brain region, the mean CoV is expected to be around 7.7–9.9% for whole-brain normalized images (20). In our study, the CoV across all PET global mean-normalized images was similar (11.61 ± 1.41%). However, as seen in our histogram data, glucose uptake alterations in pathological situations might induce bias when using this normalization method. This might lead to underestimation of the normalization factor and visualization of unspecific regions of altered glucose uptake (8).

The disadvantages of the abovementioned methods have led to the development of new data-driven methods of intensity normalization, which are thought to induce less bias during PET analysis (8, 21–23). In the histogram-based intensity normalization method, ratio images are generated by voxel-wisely dividing coregistered PET images by a normal database template. When histograms of these ratios are generated, the maximum of the histogram that represent the most prevalent ratio is chosen as normalization factor. In our study, histograms of [18F]-FDG images post-ICH showed a higher prevalence of lower ratios (0.4–0.8) than the histograms of normal [18F]-FDG rat brain scans. As mentioned above, these lower values in the image might have an effect on the global whole brain uptake and could thus induce bias when using normalization methods based on global mean scaling. The most prevalent values in the histograms, which were centered around 1, were unaffected by these lower ratio values, demonstrating that the histogram-based procedure might be a more robust method of intensity normalization. The much lower CoV (6.71 ± 2.05%) found after histogram-based normalization across normal [18F]-FDG rat brain scans as compared to global mean scaling or no normalization, also indicates that our results are influenced to a much lesser extent by intersubject variability when using this intensity normalization method.

Voxel-wise statistical comparisons between [18F]-FDG PET scans of ICH animals and the normal rat brain atlas clearly showed a region of decreased PET signal at the site of the lesion. Both the group-level and individual-level analysis methods proved to be feasible after histogram-based intensity normalization, since no clear regions of significantly altered glucose uptake were found when comparing [18F]-FDG PET scans of control animals to the normal database.

Although this study is situated in the preclinical field, the methodology and analysis procedures are translatable to clinical research. In this study, a workflow of PET atlas generation was proposed using a recent intensity normalization technique, yielding good results. In the future, it might be advantageous to construct gender- and age-specific atlasses as a way to ameliorate diagnosis, prognosis, and response evaluation in the clinic. Similarly, gender-, age-, and strain-specific atlasses for different animal models could be developed in a preclinical setting. In this way, individual as well as group analyses of pathological PET alterations compared to normal [18F]-FDG uptake, could be tailored to the biological characteristics of the subjects in question.

There are some limitations to this study. First, only male rats were used in the experimental procedures. Thus, the results of this study cannot be generalized to female rats. This choice was made because of the potential effects of the estrous cycle on PET changes in female rats (24, 25). As stated above, experimental as well as clinical research could benefit from sex-specific normal PET atlasses for subsequent analysis. Second, there was some variability in injected [18F]-FDG dose (32.5 ± 7.8 MBq) due to variations in daily [18F]-FDG availability in our facility. However, before any normalization method was performed, PET voxel-values in kBq/cc were converted to SUVs as a correction for injected dose and animal weight.

In conclusion, histogram-based intensity normalization is a useful data-driven technique for PET normalization and reduces the inter-subject variability compared to the commonly used global mean scaling method and no normalization. This approach is suitable for voxel-wise statistical analysis of glucose uptake changes compared to a normal brain atlas, on an individual as well as on a group level. This work highlights the importance of the harmonization of intensity normalization of [18F]-FDG PET images for subsequent voxel-wise analysis.

## Data Availability Statement

The raw data supporting the conclusions of this article will be made available by the authors, without undue reservation.

## Ethics Statement

The animal study was reviewed and approved by the Ethical Committee on Animal Experiments of Ghent University.

## Author Contributions

SP, RR, BD, PB, VD, and CV: study design. SP: experimental work and data collection. CG: development of animal model. SP, RR, EC, VD, and CV: data analysis. SP, RR, VD, and CV: manuscript preparation. All authors manuscript review.

## Funding

SP was supported by the Ghent University Special Research Fund (BOF, 01D27418). RR was supported by the BOF, CG, and EC were supported by the Research Foundation Flanders (FWO, 1181719N and 1S90218N). PB was supported by the FWO, BOF and Ghent University. VD was supported by Ghent University.

## Conflict of Interest

The authors declare that the research was conducted in the absence of any commercial or financial relationships that could be construed as a potential conflict of interest.

## Publisher's Note

All claims expressed in this article are solely those of the authors and do not necessarily represent those of their affiliated organizations, or those of the publisher, the editors and the reviewers. Any product that may be evaluated in this article, or claim that may be made by its manufacturer, is not guaranteed or endorsed by the publisher.
